# The Diagnosis of Diseases of the Rectum and Anus

**Published:** 1909-07-10

**Authors:** P. Lockhart Mummery

**Affiliations:** Senior Assistant Surgeon to St. Mark's Hospital for Diseases of the Rectum; and to the Queen's Hospital


### HOSPITAL CLINICS.

### THE DIAGNOSIS OF DISEASES OF THE RECTUM AND ANUS.

By P. LOCKHART MUMMERY, F.R.C.S., Senior Assistant Surgeon to St. Mark's Hospital for Diseases of the Rectum; and to the Queen's Hospital.

(A Lecture delivered at the Polyclinic, June 14, 1909.)

GENTLEMEN,—The subject of my lecture is the Diagnosis of Diseases of the Rectum and Anus; and I propose to proceed from the point of view of how one ought to tackle a case presenting itself with doubtful disease of that part.

The first point is the absolute necessity of making an examination. It is scarcely believable, but I assure you many people diagnose diseases of the rectum and anus simply from the symptoms. The reason is fairly obvious. The patient is apt to walk into your consulting room, saying, "I have got piles, I wish you would give me some ointment for them"; and he does not want to take his trousers down; and in the case of a lady she does not want to undress so that there can be an examination. And it must be said that in some cases the doctor is too careless to make an examination. To treat any case of doubtful disease of the rectum without making an examination is iniquitous, because it often results in most grave conditions having been missed.

I have frequently seen cases in hospital, with a note from the doctor saying "I have been treating this case for some months for piles, but it does not seem to be getting better." I examine, and perhaps find a carcinoma, and inquiry shows that the doctor has not even troubled to put his finger inside the rectum; if he had done so he could not have missed such an obvious condition. And in many cases the patient has had to have colotomy performed, whereas if the disease had been detected when the doctor first saw the case excision of the cancer might have been possible. It is wrong to treat any case, however apparently trivial, without making an examination of the rectum.

The value of symptoms in diseases of the rectum is practically negligible. My experience is that if you try to diagnose diseases of the rectum by symptoms you will be wrong in 50 per cent. of the cases. The patient with fissure will detail the symptoms of carcinoma, and the patient with carcinoma will complain of symptoms which seem to point to fissure, or of mere pruritus.

It is advisable to ask the patient questions, and I always start by making notes concerning the history, because by so doing you give the patient some confidence. It is a mistake when your patient comes into the consulting room to put him at once on the sofa and commence to examine his rectum. Moreover, by talking to him for a few minutes you can get a good idea of what sort of person you are dealing with. It is very desirable to get this confidence before proceeding to the examination. The difficulty is to get to know what it is exactly that the patient complains of; patients often wander off into all sorts of side remarks which have nothing to do with the matter. I ask whether it is pain, and if so, whether it keeps them awake at night, or whether it comes on after the bowels have acted. Or is it bleeding, and if so, how much? The best thing is to get them to compare it with something that they know. One patient with a quite trivial lesion will complain of pain which another would not bother about; a healthy man with the same condition would say there is nothing to bother about, though examination will show he has considerable disease. Ask whether the pain interferes with the patient's ordinary vocation, whether it is worst on standing, or on lying down, whether it keeps him awake at night, or makes him perspire. The neurotic woman will often complain of pain when she is not suffering any at all.

With regard to the method of examination, the first important point is the position of the patient. There are two positions in which to examine patients with rectal disease. The first, and the more universally used, is the left "Sims" position; that is to say, the position on the left side. This is far the better, because it enables you to use the right hand. Ask the patient to lie on the couch, on the left side, draw the knees well up, and put the left elbow under the chest. If the patient is a lady, wearing a big hat, I put a couple of pillows under the head, and let the hat hang over the edge of them, out of the way. The other position is what is called the "knee-elbow" position. The knees should be separated, and the patient should rest on the knees and elbows; the important thing in this posture is to get the thighs square to the couch. It has its disadvantages; it is not a nice position to ask ladies to assume, and if you have to do an operation, it is an uncomfortable position for the patient to occupy for more than a few minutes. In most circumstances the left Sims position is to be preferred.

A good light is essential, and by far the best method is to use artificial light, by means of an electric forehead lamp, with a 4-volt Osram lamp, worked from a little battery or from a rheostat on the wall. If you always use the same light, you get accustomed to the colours presented. And you can throw this light down a speculum or other narrow cavity better than you can daylight, even when that is good. Vaseline is the best material to use as a lubricant, but there are certain cases in which its use is not advisable, such as pruritus, or external piles, where you may want to paint it with some lotion, or where you want to use cocaine, or clean it for an operation. Vaseline is of no use then, as you will waste ten minutes in getting it off. For those cases I do not think anything is better than ordinary ether soap, and if you want to wash this off, you can do it with a little cotton wool and water.

First, look at the appearance of the parts, because the appearance of the parts outside will often give you a very useful guide as to what is likely to be seen. Examine the skin round the anus for fissures, cracks, and eczema, or for the results of pruritus; in the latter case the skin looks like wet washleather, and slightly moist. One also looks for alterations of contour. Then one separates the anus somewhat to see if there are fissures or piles. A fissure can generally be seen by carefully separating the walls of the anus.

Digital examination requires doing carefully, and some experience of making a digital examination is valuable; because, in the first place, a person who has not had experience of examination of the rectum will not always recognise the lesion when he puts his finger into the rectum; and secondly, you want to make the examination without hurting the patient. I often have men up at St. Mark's to make rectal examinations. Frequently one will see a patient, who has not made any complaint when the house-surgeon or I have made an examination, when the visiting doctor puts his finger into the rectum, nearly jumping off the couch in agony. But, except in the case of fissure, this examination can be made satisfactorily without in the least hurting the patient.

First, be sure to have the finger well lubricated, and, needless to say, the nails should be cut quite short. Then introduce the finger quite slowly. Put the flat of your finger on the edge of the anus, then tip it up slowly, keeping up a steady pressure. Again, never move your finger quickly in the anus, and do not screw it round, except very slowly; sudden movements cause the sphincter to contract violently, and not only hurt the patient, but prevent your getting your finger in. A mistake often made by those who are not used to this sort of thing is that they will often feel too high up for the disease. The majority of lesions in the rectum which one wants to feel are quite low down, within three-quarters of an inch of the orifice. Piles you cannot feel, because they are not feelable.

One next puts the thumb on the outside, and feels for indurated patches between one's fingers. In this way one feels for ischio-rectal abscess. Then pass the finger into the anus, and feel for growth high up. One can use indiarubber cots, but I only do so where there are fistulæ, carcinoma, or reason to suspect syphilitic disease. And if a patient has purulent proctitis one does not want to put one's fingers into the pus. These cots spoil the sense of touch to some extent.

With regard to probes and bougies, probes are not required. A probe will only pass along a straight channel, and the skilled finger can appreciate a fistula under the skin better than it can be appreciated by a probe. Moreover, the passage of a probe along a fistula is painful. A probe is not necessary in fistula until one comes to the operation. Bougies and things of that sort, which were much used some time ago when examining for strictures in cases of doubtful carcinoma and fibrous stricture, are now entirely replaced by the sigmoidoscope; they will not give anything like the same information as will this instrument. With regard to specula, three-fourths of them are absolutely useless.

There are two desiderata about a speculum (1) to be able to see the parts properly, and (2) to use it without hurting the patient. One surgeon in London who does this sort of work says no speculum should be used in the rectum without giving the patient an anæsthetic. That may have been true six or seven years ago, but it is not true now, because some of the modern speculæ can be used, with care, quite easily on the most nervous patient without hurting him, except in certain cases where there is a fissure or in tubercular ulceration, where there is very great pain. The worst kind of speculum is the so-called bi-valve, which is really a diabolical form. You must pass it with the edge against the lesion which you want to see, and you push the edge of the speculum against the sore place, and when you open the speculum you will tear the sore place open, and the average patient will scream. The withdrawal of the speculum is frequently very difficult, because the mucous membrane will prolapse; I have known of one case in which an anæsthetic had to be given for the withdrawal of the speculum. Any form of bi-valve speculum should be done away with. Another form of speculum is the fenestrated—that is to say, with a gap in the side. The ordinary form is a hollow, with a piece cut out on one side; mucous membrane will prolapse into the instrument, and the withdrawal of it will usually cause very great pain.

The speculum I show you has got very broad edges and a sloping end, and it is short and slightly conical, so that one can see easily. If it is well lubricated, and passed slowly, it can be used on anybody, even in cases of fissure. The only part which has got to pass the lesion is a narrow bit at the extreme end, and that is smooth, and does not hurt. If you want to examine all round an anus, there is a temptation to turn the instrument, but you should avoid that: take the instrument out and replace it where you want it. It is very useful for examining such things as fissure, openings into fistulæ, ulcerations, etc., which occur in the first three-quarters of an inch from the anus.

Remember, you cannot feel a pile; unless it is thrombosed or congested, the ordinary pile is a loose fold of membrane, though every loose fold of mucous membrane is not a pile. You can only see piles, and you must get the patient to strain down as you remove your finger, and then you can sometimes get a pile to come down on to your finger. It is better to see the pile *in situ*, and for that purpose this instrument is the best I know; it is a modification of Kelly's; his was a conical-shaped tube, and had a sharp edge, so you had to be careful never to push it in, or you cut the mucous membrace. This has a very broad edge, and parallel sides. With it one can easily see piles, and examine for proctitis, and though it is not comfortable, it does not hurt the patient if it is passed carefully. After smearing the whole thing all over with vaseline, I hold the handle of the obturator against my fingers, and press it gently against the sphincter, telling the patient in the meanwhile to relax the sphincter as much as possible. Usually it will slip in easily, and when once it is in you can remove the obturator.

If you examine a man, especially an elderly man, who may have an enlarged prostate, it is important to keep the point backwards, because otherwise the end will impinge against the prostate as it goes in, and will cause discomfort. You can look through it and see what the condition of the mucous membrane is. Then you can withdraw it, and push it back again, and that will bring another portion of mucous membrane into view. Those two specula are sufficient to examine any cases of rectal disease not higher up than two or three inches.

With these two instruments one can examine any lesion two inches from the anus. For carcinoma, ulcerative colitis, or disease in the sigmoid flexure, one needs to use the sigmoidoscope, which is of extraordinary value in these cases. There are many cases of rectal disease which should be examined with the sigmoidoscope, in addition to specula, because even if a patient has piles, it is important to find out whether there is also a lesion higher up. In a case of pruritus, it is necessary to find out whether he has had proctitis. It should be realised that sigmoidoscopic examination can be made without an anæsthetic; I commonly so use it, and do not cause any more discomfort than with a speculum. The patient has no idea what length instrument is being passed. The only sensitive part of the bowel is the last three-quarters of an inch; higher up, the bowel is insensitive, so long as you do not stretch the mesentery, which can be avoided by passing the instrument carefully. This instrument requires some practice in using; otherwise you will not be able to pass it, and know what you are looking at; and also because you can do damage with it unless you are careful. It has an obturator. One greases the whole thing, and passes it with the obturator in place. The object of the obturator is simply to pass it through the sphincter; the obturator should then be removed, and should not be put back again. The electric light is then passed into the instrument, and the inflator attached. After that, the instrument is passed entirely by sight; the rectum is opened out in front of it by puffs of air. I advise people not to push it past a fold, but to blow the fold out of the way. After some experience you can use the end of the instrument to help you to get round a fold.

In ulcerative colitis no one should attempt to use the instrument unless he is very experienced, because the bowel wall is very rotten, and roughness may result in perforation. I have passed it in cases where the bowel wall has been so thin that I could see the coils of bowel through the wall, but no injury has resulted. To work this instrument, I at first used to have big batteries, but now I use this small accumulator, which has no liquid acid to run out. There is no rheostat on it; it is four-volts, and will light the lamp for seven or eight hours. The secret of it is using Osram lamps, which remain quite cold. I show you also an irrigating sigmoidoscope; also one with a much larger bore, so as to pass instruments down it. But that must only be passed with an anæsthetic. As an alternative to a battery, there is a rheostat, an American instrument, with graphite resistance. This cuts down a circuit of 240 volts to work a 4-volt Osram lamp, and it is very satisfactory.

The sigmoidoscope is of such value that everyone should be accustomed to using it who deals with diseases of the bowel. I had a patient from the Midlands the other day who had been treated for fifteen months for colitis. He was forty-one years of age, and had had occasional diarrhœa. He passed some mucus at times, but had never seen blood. If he ate too much or stood a long time he had a colicky pain in the abdomen. I found the rectum and lower part of the sigmoid flexure normal. Then on passing the sigmoidoscope to its full length I found a cancerous ulcer about the size of half a crown in the centre of the sigmoid flexure, which accounted for his symptoms. Though the abdomen was easy to examine, I could detect nothing in that way; and without this instrument it would have been quite impossible to diagnose that growth for another four or five months, by which time it would probably have been inoperable. It is now excellent for excision and anastomosis.

Examination of the abdomen must be done in any case. If there are piles, the patient may have cirrhosis of the liver, or some other condition to which piles are secondary, and you must not treat the piles if there is some cause in the abdomen causing them. Whatever the condition of the rectum may be, it is always advisable to pass the finger into the rectum and examine as high as possible; otherwise you may fall into error, and miss carcinoma higher up.

I have brought for your inspection models to illustrate the more common conditions of the anus. Let us take them from the standpoint of symptoms.

The most common condition which results in pain is fissure. The diagnosis is fairly easy. By passing the finger into the anus you will feel the hard indurated walls. Another cause of pain is strangulated piles. There is swollen mucous membrane at the edge, and partial obstruction of the blood supply due to nipping of the piles by the sphincter.

Another cause of pain is external piles; they look like purple grapes at the edge of the anus; they are tender and fairly hard. The exact appearance will depend on the stage at which they are seen; sometimes their surface will have become ulcerated and gangrenous. In pruritus, with eczema, there is a whitish appearance of the surrounding skin, with deep excoriated fissures and reddish eczema round the edges; this is a very characteristic result of pruritus.

Sometimes part of the bowel prolapses, and it comes down as a painful red blob, and is occasionally mistaken for piles. It is very important to have some idea of what secondary syphilis of the anus looks like; as it is not advisable to put one's finger on to condylomata, you must examine those with the finger stall. Sometimes they are very deceptive, and one can examine them digitally without knowing what they are. If that is so, the hand should be thoroughly sterilised afterwards.

Epithelioma is well illustrated by this model: an indurated ulcer spreading on to the skin. Tuberculous ulceration is a very characteristic condition; there is a shallow, callous-looking ulcer, with undermined blueish edges, and spreading out in different places of the anus, very often at the same time.

A much rarer form is the lupoid condition shown by this model. The first twice I saw it I mistook it for early epithelioma.

